# Diagnostic value of galectin-3, fractalkine, IL-6, miR-21 and cardiac troponin I in human ischemic cardiomyopathy

**DOI:** 10.18632/aging.205953

**Published:** 2024-06-18

**Authors:** Le Wang, Min Li, Mingqi Zheng, Yida Tang, Zhiyu Yang, Guoping Ma, Qinghou Zheng, Liu Li, Yu Wang, Fangfang Ma, Gang Liu

**Affiliations:** 1 Department of Cardiology, The First Hospital of Hebei Medical University, Shijiazhuang, Hebei, China; 2 Hebei Key Laboratory of Heart and Metabolism, Shijiazhuang, Hebei, China; 3 Hebei International Joint Research Center for Structural Heart Disease, Shijiazhuang, Hebei, China; 4 Hebei Key Laboratory of Cardiac Injury Repair Mechanism Study, Shijiazhuang, Hebei, China; 5 Hebei Engineering Research Center of Intelligent Medical Clinical Application, Shijiazhuang, Hebei, China; 6 Department of Cardiology, Peking University Third Hospital, Beijing, China

**Keywords:** ischemic cardiomyopathy, galectin-3, fractalkine, interleukin-6, microRNA-21, cardiac troponin I

## Abstract

Objective: The primary objective of this study was to assess the diagnostic potential of galectin-3 (Gal-3), fractalkine (FKN), interleukin (IL)-6, microRNA(miR)-21, and cardiac troponin I (cTnI) in patients with ischemic cardiomyopathy (ICM).

Method: A total of 78 ICM patients (Case group) and 80 healthy volunteers (Control group) admitted to our hospital for treatment or physical examination from Aug. 2018 to Feb. 2020 were included in the current study. The serum concentration of Gal-3, FKN, IL-6, miR-21, and plasma expression of cTnI of both groups were determined. The severity of ICM was classified using New York Heart Association (NYHA) scale.

Results: When compared with the control group, the case group had a significantly high blood concentration of Gal-3, FKN, IL-6, miR-21, and cTnI (*P* < 0.001). NYHA class II patients had lower blood levels of Gal-3, FKN, IL-6, miR-21, and cTnI than that in patients of NYHA class III and IV without statistical significance (*P* > 0.05). However, statistical significance could be achieved when comparing the above-analyzed markers in patients classified between class III and IV. Correlation analysis also revealed that serum levels of Gal-3, FKN, IL-6, miR-21, and cTnI were positively correlated with NYHA classification (R = 0.564, 0.621, 0.792, 0.981, *P* < 0.05).

Conclusion: Our study revealed that up-regulated serum Gal-3, FKN, IL-6, miR-21, and cTnI levels were closely related to the progression of ICM. This association implies that these biomarkers have diagnostic potential, offering a promising avenue for early detection and monitoring of ICM progression.

## INTRODUCTION

Ischemic cardiomyopathy (ICM), a prevalent clinical entity, significantly contributes to cardiovascular mortality, particularly as the aging population witnesses a rising incidence, heightening public health concerns [1]. This condition accounts for 20% of coronary artery disease, with a 5-year mortality rate ranging from 50% to 84%, placing it at the forefront of global cardiovascular research. A comprehensive understanding of ischemic heart disease’s pathophysiology is pivotal for effective prevention and therapy [1]. Gal-3, a biomarker in heart failure diagnosis and proposed for general population prediction, has limited research in ischemic heart disease [2]. Fractalkine (FKN), an irregular chemokine, has primarily been studied for its role in myocardial infarction and fibrosis, but its association with ischemic heart disease remains underexplored [3]. IL-6, linked to myocardial fibrosis and remodeling, has been implicated in dilated cardiomyopathy, yet its connection to ischemic heart disease is scant [4]. miR-21, a member of the miRNAs family, is commonly expressed in various tissues and has shown abnormal expression in multiple cardiovascular diseases, influencing their progression [5]. However, its implications in ischemic heart disease remain uncharted. Cardiac troponin I (cTnI), a cardiac-specific protein, enters circulation following myocardial ischemia or necrosis, triggering immune responses and generating persistent anti-cTnI autoantibodies [6]. Reports on cTnI’s involvement in ischemic heart disease are scarce.

Therefore, the current study aims to delve into the potential applications and prognostic significance of Gal-3, FKN, IL-6, miR-21, and cTnI in ischemic heart disease patients, with the goal of advancing clinical management strategies.

## METHODS

### Clinical data

78 ICM patients (Case group) admitted for treatment and 80 healthy volunteers (Control group) admitted for physical examination to our hospital from Aug. 2018 to Feb. 2020 were involved in the current study. Patients in Case group were composed of 48 males and 30 females, aged from 46 to 68 with a mean age of 53.38 ± 6.28; volunteers in the Control group were composed of 49 males and 29 females, aged from 44 to 69 with a mean age of 53.65 ± 6.31. Parameters like gender and age were comparable between the two groups (*P* > 0.05). The study was approved by the ethics committee of the First Hospital of Hebei Medical University and adhered to the Declaration of Helsinki. All patients gave written informed consent for participation and publication of data. General parameters like the medical history and smoking history of participants were recorded.

### Inclusion and exclusion criteria

Inclusion criteria of this research were: (1) patients involved should be diagnosed with ICM according to Chinese Guidelines for the Diagnosis and Treatment of Heart Failure; (2) at least one major coronary stenosis >50% as determined by coronary angiography; (3) reperfusion injuries could be detected at coronary artery distribution by cardiac emission computed tomography; (4) myocardial late enhancement emerged consistently in coronary artery distribution as determined by cardiac magnetic resonance; (5) patients with New York Heart Association (NYHA) classification graded larger than II.

The exclusion criteria were: (1) participants with autoimmune disease, cerebrovascular diseases, malignant tumor, acute coronary syndrome, idiopathic cardiomyopathy, other dilated cardiomyopathy, or other vascular diseases; (2) participants with rupture of mitral chordae tendineae, dysfunction of papillary muscle or ventricular septal rupture; (3) participants with other severe ICM complications; (4) participants with inadequate medical history of ICM; (5) participants with hepatic or renal function deficiency or complicated with chronic/acute infection.

### Serum collection and protein determination

3–5 ml morning fasting elbow vein blood was collected and centrifuged at 3000 rpm for 10 minutes. The serum was collected for further determination. Serum Gal-3 (purchased from Jiangsu Jianglai Biotechnology Co., Ltd.), FKN (purchased from Beijing Lvyuan Bode Biotechnology Co., Ltd.), IL-6 (purchased from Shanghai Sangon Biotechnology Company Limited), and cTnI (purchased from Shanghai Haling Company Limited) were determined by enzyme-linked immunosorbent assay (ELISA).

### Detection of miRNA-21 expression

The relative expression of miRNA-21 was determined via a real-time PCR (RT-PCR) method [7]. Total RNA was isolated from whole blood by using miRNeasy Seum/Plasma Kit (Qigen, Germany) as instructed by the manufacture. Isolated mRNA was then undergone quality control by measuring the absorbance at 260 nm. A cDNA library was generated by using a reverse transcription kit followed by the manufacture’s instruction (Fermentas, France). Each RT-PCR reaction was conducted in a 10 μl reaction system containing 1 μg cDNA, 5μL SYBR Green PCR Mix, 1 μL Primer, and RNase-free water. The reaction condition is 95°C 15 min, 40 cycles at 94°C 15 s, 55°C 30 s, 70°C 30 s. Relative miR-21 expression was first normalized to U6 expression by the ΔΔCt method and then normalized the value for each sample to the first sample from the Control group. Primer sequences used are as following: miR-21, 5′-TAGCTTAT-CAGACTGATGTTGA-3′; U6, 5′-AT-TCGTGAAGCGTTCCATATIT-3′.

### Statistical analyzes

Statistical analyzes were performed by using SPSS 21.0. Continuous variables were presented as mean ± standard deviation and differences between the two groups were calculated by Student’s Independent *T*-test. Logistic data were presented as percentage (%) and differences between the two groups were calculated by χ2 test. The distributions of quantitative data were assessed by using SPSS. Differences between two groups were analyzed by student *t*-test. Comparison among three groups were performed by using one-way ANNOVA, and the within-group variation were identified by using the LSD method. Correlation analyses were performed by using Spearman correlation analysis. A *p*-value of < 0.05 was considered to be statistically significant.

## RESULTS

### Demographic data

General parameters, including age, gender, body mass index (BMI), smoking or not, of participants from two groups, were comparable (*P* > 0.05). However, participants in the case group had a significantly high level of blood pressure, high occurrence rate of diabetes and dyslipidemia, and higher NYHA grade than that of participants from the control group, as presented in Table 1.

**Table 1:** Demographic data.

**Parameters**	**Case group (78)**	**Control group (80)**	**χ^2^/t**	***P*-value**
Age	53.38 ± 6.28	53.65 ± 6.31	−0.270	0.788
Male (*N*)	48	49	0.027	0.869
BMI (kg/m^2^)	25.41 ± 2.03	25.27 ± 1.03	0.549	0.587
Smoking	31 (39.74)	28 (35.00)	0.380	0.538
Hypertension (*N*)	34 (43.59)	−		
dyslipidemia (*N*)	33 (42.71)	−		
Diabetes (*N*)	19 (24.36)	−		
Systolic blood pressure (mmHg)	133.98 ± 12.28	110.89 ± 13.91	11.051	0.000
Diastolic blood pressure (mmHg)	81.03 ± 3.28	65.92 ± 3.29	28.906	0.000
NYHA classification (*N*)				
Class II	30 (38.46)	−		
Class III	37 (47.44)	−		
Class IV	11 (14.10)	−		

### Concentration or expression of Gal-3, FKN, IL-6, miR-21 and cTnI

**Figure 1:**
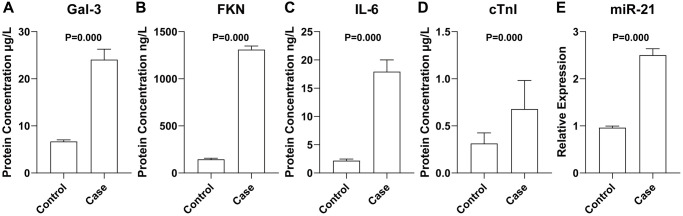
Concentration or expression of Gal-3 (**A**), FKN (**B**), IL-6 (**C**), cTnI (**D**), and miR-21 (**E**). Data were presented as mean ± standard deviation and differences between the two groups were calculated by Student’s Independent *T*-test. *N* = 78 for each group.

As presented in Figure 1, participants in the case group had a significantly higher level of Gal-3, FKN, IL-6, miR-21, and cTnI than that of participants from the control group (*P* < 0.0001).

### Level of Gal-3, FKN, IL-6, miR-21 and cTnI in different NYHA grades

As presented in Table 2, the blood levels of Gal-3, FKN, IL-6, miR-21, or cTnI in NYHA class II patients were lower than that in patients of NYHA class III and IV without statistical significance (*P* > 0.05). However, statistical significance was achieved when comparing those markers in patients classified between class III and IV.

**Table 2:** Level of Gal-3, FKN, IL-6, miR-21 and cTnI in different NYHA grades.

**Markers**	**Class II**	**Class III**	**Class IV**	***P*-value**
Gal-3 (μg/L)	18.92 ± 2.21^*#^	20.98 ± 2.73^#^	25.01 ± 2.63	0.000
FKN (ng/L)	1187.93 ± 34.92^*#^	1298.87 ± 35.87^#^	1428.82 ± 56.29	0.000
IL-6 (ng/L)	13.28 ± 3.29^*#^	16.98 ± 2.09^#^	19.63 ± 2.09	0.000
CTnI (μg/L)	0.53 ± 0.09^*#^	0.65 ± 0.12^#^	0.79 ± 0.27	0.000
miR-21	6.02 ± 0.27^*#^	7.68 ± 0.28^#^	9.03 ± 0.32	0.000

^*^Compared with class III; ^#^Compared with class IV.

### Correlation of Gal-3, FKN, IL-6, miR-21 or cTnI with NYHA grades

As determined by Spearman correlation analysis, the blood levels of Gal-3, FKN, IL-6, miR-21, or cTnI were found to be positively correlated with NYHA grades (Table 3, R = 0.564, 0.621, 0.792, 0.981, respectively, *P* < 0.0001).

**Table 3:** Correlation of Gal-3, FKN, IL-6, miR-21 or cTnI with NYHA grades.

**Markers**	**NYHA grades**	
	**R**	***P*-value**
Gal-3	0.564	0.000
FKN	0.621	0.000
IL-6	0.792	0.000
miR-21	0.981	0.000
CTnI	0.813	0.000

Data was analyzed by using Spearman correlation analysis.

## DISCUSSION

ICM, a syndrome characterized by persistent myocardial ischemia and oxygen deficiency due to coronary atherosclerosis, is marked by diffuse fibrosis, compromising cardiac function and presenting with symptoms such as angina, heart failure, and arrhythmias. As a distinct subtype of coronary artery disease, its pathophysiology is rooted in atherosclerotic plaques. As a significant contributor to heart failure, ischemic heart disease has experienced a growing prevalence, yet lacks a standardized clinical diagnostic approach. Diagnoses primarily rely on subjective patient reports, physical examination, and imaging studies, lacking robust, high-specificity, and high-sensitivity laboratory tests for prompt and accurate identification. Consequently, ischemic heart disease has garnered substantial attention in recent clinical research efforts. This study endeavors to explore the utility and prognostic significance of Gal-3, FKN, IL-6, miR-21, and cTnI in the context of ICM patients.

Growing evidence has proved that inflammatory reaction was closely related to the pathogenesis of ICM. Laboratory examination of blood inflammatory markers for diagnosis of other cardiovascular diseases has been widely adopted because of its features like cost-efficient, convenient, practical, and easy to adopt [8, 9]. Gal-3, a macrophage secreting β-galactoside-binding lectin, is a powerful inflammatory factor and could induce fibrosis in multiple organs by exerting its role in neuroendocrine activation and pressure overload. Previous research has proved that serum Gal-3 is positively correlated with the pathogenesis of myocardial fibrosis and cardiac remodeling [10]. Other research also proved that Gal-3 could induce the differentiation of monocyte to macrophage and could further promote macrophage in the massive secretion of proinflammatory cytokines and chemokines [11]. Further clinical research revealed serum expression of Gal-3 was closely related to the prognosis of acute decompensated heart failure [12] and the dynamic changes of Gal-3 could be used as a prognostic marker for acute decompensated heart failure with better efficiency than B-type natriuretic peptide (BNP) and N-terminal (NT) pro-BNP. Therefore, in 2010, the U.S. Food and Drug Administration has approved Gal-3 as a marker for short-term prognosis in patients with heart failure [13]. FKN, a 373-amino-acid type 1 transmembrane protein, could promote the release of vascular endothelial growth factor (VEGF) and transforming growth factor-α (TGF-α) through signal transduction via direct interreacting with its specific receptor CX3CR1. Those factors were known to be involved in the process of cardiac injury and repair and cardiac fibrosis. Reports have demonstrated that the serum level of FKN was closely related to the pathogenesis of coronary heart disease [14]. Also, FKN antagonists were reported to have the ability to decrease myocardial infarction size, improving myocardial remodeling, and cardiac function in an animal model of myocardial infarction [15]. Further *in vivo* study found that the anti-fibrotic effect of FKN antagonist was achieved by inhibiting apoptosis of cardiomyocytes and by promoting the proliferation of cardiac fibroblasts. IL-6, a member of the interleukin family with multiple functions, could be secreted by monocytes, T cells, B cells, macrophages, and endothelial cells. By bind to its receptor, IL-6 mediated signal transduction could be widely activated in the cardiovascular system, neural system, immune system, and endocrine system. The report has found that IL-6 participates in the pathogenesis of cardiac remodeling, and cardiac fibrosis. As demonstrated by Li et al., in ICM, IL-6 overexpression could result in imbalanced matrix metalloproteinase expression via mitogen-activated protein kinase-related signal transduction [16], thus promoting the release of collagen I and III, and ultimately promote the progression of cardiac remodeling.

The prevalence of cardiovascular disease is intricately related to multiple mechanisms, with recent studies highlighting the pivotal role of epigenetic changes, especially changes of miRNAs, in the progress of cardiovascular disease. Previous research has confirmed miRNAs could be widely found in animals and plants and could be related to cell hypertrophy, fibrosis, and myocardial development. As miRNAs can participate in many physiological and pathological processes by regulating corresponding target genes, they may become novel targets for the treatment of ICM [17]. miR-21 is one of the most well-studied miRNAs in the pathology of cardiovascular disease [18]. cTnI is a substance released into the blood when myocardial cells are damaged, and it is currently one of the important markers for diagnosing myocardial damage. As reported by Luo et al. [19], cTnI level in patients with septic cardiomyopathy has a significant trend of increase, and this cTnI increase was found closely related to the prognosis of septic cardiomyopathy. Our results have found that blood levels of Gal-3, FKN, IL-6, miR-21, and cTnI were significantly upregulated in ICM patients. Additionally, the level of those markers grows with grades of NYHA classification, suggesting those markers may participate in the pathogenesis of ICM.

A significant upregulation of Gal-3 was previously observed in atherosclerotic tissues of rats [20]. And Gal-3 is highly expressed in the unstable plaque area of the human carotid artery with the function of promoting local inflammation, which might potentially promote the occurrence and development of coronary atherosclerosis [21]. FKN is highly expressed in vascular injury and atherosclerotic plaques, which may be closely related to platelet activation and adhesion, further suggested that FKN may promote thrombus formation [22], FKN can participate in the damage of blood vessels by strengthening the adhesion function of monocytes and endothelial cells. Also, FKN can promote the proliferation of human smooth muscle cells and inhibit their apoptosis, and can induce vascular lumen stenosis [23]. Reports have found that IL-6 is involved in the pathogenesis of coronary heart disease and is closely related to the severity of coronary artery disease with a higher level of IL-6 related to severe conditions of patients with coronary heart disease [24]. Toldo et al. [25] have found myocardium-specific over-expression of miR-21 could significantly reduce ischemia area and symptoms of heart failure via Fas ligand (FasL) signal pathway when compared with cardiac ischemia-reperfusion wild type mouse. This result suggested that miR-21 has a cardiac protective role by reducing myocardial cell apoptosis induced by myocardial ischemia-reperfusion so that it could restrain the ventricular remodeling process. Saiki et al. [26], found that increased serum cTnI level in patients with acute heart failure was closely related to prognosis. Other research also found that serum cTnI level is positively correlated with the condition of patients with acute heart failure with higher serum cTnI level represents more severe the condition [27]. Therefore, the present study further analyzes the relationship between the blood level of Gal-3, FKN, IL-6, miR-21, cTnI, and grades of NYHA classification. Our results have confirmed that blood levels of Gal-3, FKN, IL-6, miR-21, and cTnI were positively correlating with grades of NYHA classification, suggested that Gal-3, FKN, IL-6, miR-21, and cTnI might participate in the progress of ICM.

This study has several limitations. Firstly, the current study is a single-centered study with limited cases involved, further multi-centered observations with a larger cohort are essential for validating the conclusion. Additionally, the diagnostic utility of Gal-3, FKN, IL-6, miR-21, and cTnI was not evaluated in the current study due to the restricted sample size.

## CONCLUSION

Our study has uncovered that circulating concentrations of Gal-3, FKN, IL-6, miR-21, and cTnI exhibit promise as potential diagnostic markers for ICM. The current work may provide a useful referee for ICM diagnosis after massive validation in the future.
